# Comparative Efficacy of Fractional CO_2_ Laser Combined with Topical Steroid Cream versus Solution for Post-Thyroidectomy Scar Treatment: A Prospective Study

**DOI:** 10.3390/healthcare12161605

**Published:** 2024-08-12

**Authors:** Ching-Ya Huang, Yuan-Hao Yen, Cen-Hung Lin, Yueh-Ju Tsai, Ko-Chien Lin, Hui-Ping Lin, Ching-Hua Hsieh

**Affiliations:** Department of Plastic Surgery, Kaohsiung Chang Gung Memorial Hospital, Chang Gung University College of Medicine, Kaohsiung 83301, Taiwan; b101106030@tmu.edu.tw (C.-Y.H.); medik@cgmh.org.tw (Y.-H.Y.); gigilin119@cgmh.org.tw (C.-H.L.); johnnytsai@cgmh.org.tw (Y.-J.T.); kochien45@cgmh.org.tw (K.-C.L.); poppy952@cgmh.org.tw (H.-P.L.)

**Keywords:** post-thyroidectomy scars, fractional carbon dioxide laser, laser-assisted drug delivery (LADD), clobetasol propionate, triamcinolone acetonide

## Abstract

Backgrounds: Post-thyroidectomy scarring is a common illness impacting patient quality of life. Fractional carbon dioxide (CO_2_) lasers and topical steroids delivered via laser-assisted drug delivery (LADD) have shown potential for scar treatment. However, ideal steroid formulations (cream vs. solution) when combined with laser therapy remain unclear. Methods: This study included 12 patients receiving fractional CO_2_ laser on post-thyroidectomy scars. After laser treatment, one scar half received topically applied steroid cream, while the other half received steroid solution. The Patient and Observer Scar Assessment Scale (POSAS) was used to measure the scar conditions at the time prior to the first treatment and one year later by the patients themselves and by the surgeon who did the laser treatment. Scar appearance was photographically assessed at baseline and 6 months post-treatment by four blinded evaluators using scales. Results: This study discovered a modest improvement in the appearance of post-thyroidectomy scars when combining fractional CO_2_ laser treatment with either topical steroid cream or solution. Patients and treating physicians examined the POSAS scores one year after treatment found significant improvements in all aspects of the scar conditions, with high efficacy and satisfaction levels reported by patients. Conclusions: Fractional CO_2_ laser combined with topical steroid delivery, either cream or solution form, significantly enhanced post-thyroidectomy scar appearance with modest effect and high patient satisfaction. This approach may represent a promising scar management strategy along with current scar treatment for the post-thyroidectomy scar.

## 1. Introduction

Scarring following thyroid surgery can significantly impact a patient’s quality of life, affecting both aesthetic concerns and psychological well-being [1,2]. Despite advances in surgical techniques aimed at minimizing scarring, the development of visible postoperative neck scars remains a common consequence [3,4]. As a result, there is a continued demand for effective treatment modalities that can enhance the appearance and texture of these scars, aligning them more closely with the surrounding normal skin.

The management of the postoperative scar, especially in highly visible areas like the neck, poses a major challenge in post-surgical care, particularly following thyroidectomy, a common procedure that can leave prominent scars. The use of fractional carbon dioxide (CO_2_) laser treatment has emerged as a promising method to enhance the appearance and healing of these scars [5,6,7]. Fractional CO_2_ lasers are a promising technology for scar management, enabling deep penetration into the dermal layers through a matrix-style energy delivery that splits the laser beam into multiple tiny dot-like outputs. By stimulating collagen reorganization and neogenesis via photonic activation of dermal fibroblasts, this fractional method helps remove scar tissue through heat degradation and improves the texture and appearance of scars [8,9]. It also efficiently fills in depressed areas and offers a significant advancement in scar appearance [10,11].

Additionally, laser-assisted drug delivery (LADD) approaches have been shown to enhance transdermal medicine absorption [12]. Fractional lasers are used in LADD to enhance drug penetration by forming microchannels in the skin, maximizing medicine delivery while minimizing skin injury. Effective skin ablation lowers the skin’s barrier function and enhances drug absorption. Studies have demonstrated that LADD can significantly increase skin deposition and penetration of various compounds, highlighting its extraordinary capacity to increase skin permeability for better medication delivery and suggesting a potential avenue for enhanced dermatological therapies and scar management when combined with fractional CO_2_ laser treatment [12,13,14,15].

Among various strategies to mitigate scar formation, topical agents have garnered significant attention due to their non-invasive nature, ease of application, and patient compliance. Topical steroids have been extensively studied for their efficacy in reducing hypertrophic scars, characterized by an overactive healing process resulting in thick, raised scars that do not recede over time. While intralesional corticosteroids have long been the cornerstone of hypertrophic scar treatment, their administration can be painful and uncomfortable. Consequently, there has been increasing interest in topical steroids as a more convenient and less invasive alternative. These agents are thought to modulate the inflammatory response, a critical phase in hypertrophic scar formation. Studies have shown topical steroids can effectively reduce size and improve appearance by inhibiting fibroblast proliferation and reducing collagen synthesis within scar tissue [16]. Moreover, comprehensive reviews emphasize topical treatments’ potential not only for practical use but also for enhancing patient outcomes in scar management [17]. Fang et al. [18] also highlight various compounds’ effectiveness, including steroids, in managing cutaneous scars. The evidence suggests that while the therapeutic arsenal includes diverse options, topical steroids remain valuable, offering benefits and support. Moreover, different topical steroid formulations might interact differently with laser-created microchannels [19].

Combining topical steroid formulations with fractional CO_2_ lasers for LADD creates a synergistic approach that enhances the appearance of the post-thyroidectomy scar [20]. Considering that the creams and solutions may have varying viscosities and absorption characteristics influencing their ability to penetrate the microchannels created by the fractional CO_2_ laser, this study aims to assess the efficacy of fractional CO_2_ laser treatment coupled with topical steroids formulation (cream or solution) in improving post-thyroidectomy scar appearance.

## 2. Materials and Methods

### 2.1. Study Design and Patient Enrollment

Before the implementation of this study, the Institutional Review Board of Chang Gung Memorial Hospital approved the conduct of this prospective, randomized, split-body study with the reference number 201900071A3 and approval date of 3 April 2019. This study only included those patients who have undergone thyroid surgery for more than a year and have prominent hypertrophic scars. This study’s exclusion criteria encompassed patients who harbored doubts about the trial and failed to receive satisfactory answers, those deemed difficult to follow up during the study period, and those afflicted with acquired immunodeficiency syndrome, autoimmune diseases, psoriasis, or those with keloid or ulcers near the area of the scar. Finally, this study enrolled 12 patients aged 20 years or older to proceed with the study. We obtained informed consent from all participants included in this study before proceeding with this study.

### 2.2. Randomization and Split-Scar Design

The trial splitting method involved dividing each patient’s scar into two halves along the midline. A combination of the fractional CO_2_ laser and topical steroid solution was used to treat the other side, whereas one side was treated with the fractional CO_2_ laser and topical steroid ointment. The choice of which side to receive what kind of treatment was conducted using a random permuted blocks approach to ensure random assignment and balance between the two treatment options. This method employed six possible block arrangements, such as Left-Left-Right-Right, Left-Right-Left-Right, and so forth. Treatment sides were determined by drawing lots, ensuring randomization and that an equal number of patients received different treatments on the left and right sides.

### 2.3. Treatment Protocol

The patients received three monthly sessions of fractional CO_2_ laser therapy using the eCO_2_ Plus^TM^ device manufactured by Lutronic, Republic of Korea. The procedure was performed by the same surgeon, the principal investigator C.-H. Hsieh. The Food and Drug Administration granted approval for the commercial distribution of the laser device intended for dermatologic treatment. Every patient received Lidopin 5% cream (Panion & BF Biotech Inc., Taipei, Taiwan), which was applied to the scar for 30 min before each laser treatment to alleviate pain. Subsequently, we carefully eliminated the Lidopin 5% cream by using a gauze soaked with normal saline solution. Then, we treated the scar by applying a fractional CO_2_ laser twice. The laser operated at a wavelength of 10,600 nm, had a spot size of 120 mm, a pulse energy ranging from 26 to 30 mJ, a power of 30 W, and a density of 300. After the fractional CO_2_ laser treatment, a topical steroid cream (clobetasol propionate, 0.05%) or steroid solution (triamcinolone acetonide, 10 mg/mL) was evenly applied to one side and the other side scar, respectively, by gently moving a cotton swab back and forth immediately after the laser treatment. No additional application of steroid ointment or utilization of anti-scar treatments such as scar gel or silicone scar-care dressing was employed. During this study, patients were not informed which side received the cream or solution treatment. For wound care, we used neomycin ointment daily for 3–5 days postoperatively. The same physician performed treatments monthly for a total of three sessions, adhering to the same protocol and spacing each course 4 weeks apart. We took photographs before each treatment and three months after the third treatment session.

### 2.4. Management of Possible Harms 

If significant side effects such as wound formation, allergic reactions, microvascular proliferation, or worsening of pigmentation occurred, these were discussed with the patients, and further treatments may have been discontinued. The results of this study will be treated with confidentiality by the principal investigator, who will diligently safeguard the privacy of the participants. In the event that the study results are published, the identities of the participants will remain confidential, and they will be identified by numerical codes to ensure the confidentiality of their data.

### 2.5. Outcome Evaluation 

The Patient and Observer Scar Assessment Scale (POSAS) was used to measure the scar conditions at the time prior to the first treatment and one year later by the patients themselves and by the surgeon who did the laser treatment. The POSAS is a comprehensive technique used to evaluate scars, considering the viewpoints of both the patient and the physician [21,22,23,24]. The patient scale enables the patient to rate six aspects of their scar (pain, itching, color, stiffness, thickness, and irregularity) on a scale of 1 to 10. Additionally, it allows them to express their overall assessment of the scar in comparison to normal skin. The observer scale allows the clinician to provide scores for six scar-related characteristics (vascularity, pigmentation, thickness, relief, pliability, and surface area) using a 10-point scale. Additionally, it allows for an overall assessment of the scar. The POSAS was developed to address the limitations of previous scar assessment tools that did not consider the patient’s perspective, providing a comprehensive evaluation of scar quality from both the patient’s and clinician’s viewpoints. 

Additional assessment was performed by four plastic surgeons based on the photographs captured using the same camera by the same photographer (H.-P., Lin) at the time before and six months after the initial treatment. These four plastic surgeons were not involved in the laser treatment and were blinded to which therapeutics were applied to which side. The assessment of the overall scar condition included (1) the scarring condition when compared with the surrounding normal skin on a 0–10 scale, with 0 representing no difference from normal skin and 10 indicating a severe divergence, and (2) the overall scar improvement at six months after initial treatment vs. pre-treatment condition, with scores ranging from 0 to 10 and 10 indicating the most improvement. One year after the initial treatment, we collected patients’ self-reported satisfaction, which included general comments on the treatment’s efficacy and satisfaction. General efficacy was evaluated by patients’ satisfaction based on a scale from 0, which indicates no change, to 10, which indicates a substantial improvement post-treatment. Satisfaction was evaluated with a range from −10 for extreme dissatisfaction to 10 for very high satisfaction.

### 2.6. Statistical Analysis

After calculating and presenting the average values of the scales and scores from the self-assessments and evaluations before and after treatment, a Wilcoxon signed-rank test (Windows version 23.0; SPSS, Inc., Chicago, IL, USA) was used to compare the results. At *p* < 0.05, statistical significance was established. The graphs were present with a Window’s version of GraphPad Prism 6 (GraphPad Software, Boston, MA, USA).

## 3. Results

### 3.1. Comparative Outcomes in Post-Thyroidectomy Scar before and after the Treatment

**Figure 1:**
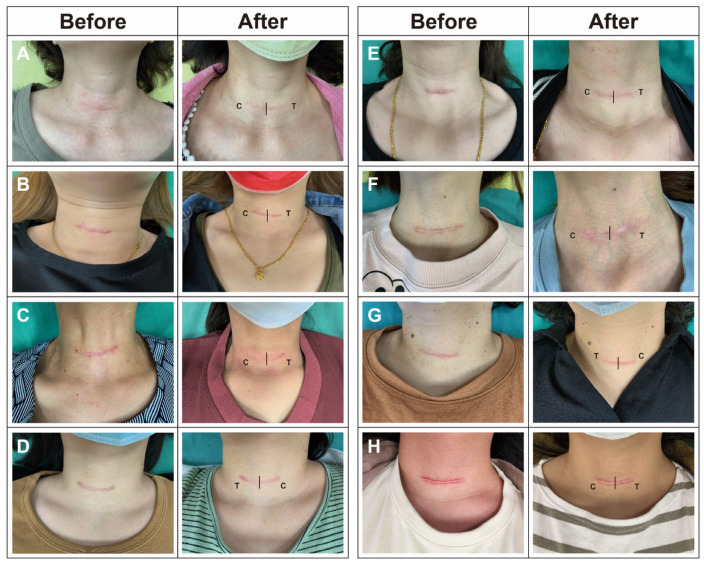
A visual comparison of four representative patients (**A**–**H**) undergoing treatment for post-thyroidectomy scars before and six months after their initial treatment. The “C” indicates sides treated with steroid cream clobetasol propionate in the concentration of 0.05%, while “T” stands for treatment with steroid solution of triamcinolone acetonide.

There were no harmful side effects discovered in this investigation, hence there was no discontinuity during the trial. This study included a total of twelve patients. Figure 1 provides a visual comparison of post-thyroidectomy scars before and after treatment on eight individuals, where each participant received treatment with either a steroid cream (“C”, indicating clobetasol propionate) or a steroid solution (“T”, indicating triamcinolone acetonide) on opposite sides. The results depicted in the figures did not show a remarkable difference between both sides treated, indicating that both treatment modalities had similar effects on the appearance of the scars. This suggests that either form of steroid application—cream or solution—could be equally effective in managing the appearance of post-thyroidectomy scars. Notably, some patients did not have a significant visual improvement in the overall scar after receiving fractional CO_2_ laser treatment (Patient H in Figure 1).

### 3.2. Outcome of POSAS Measurement 

**Figure 2:**
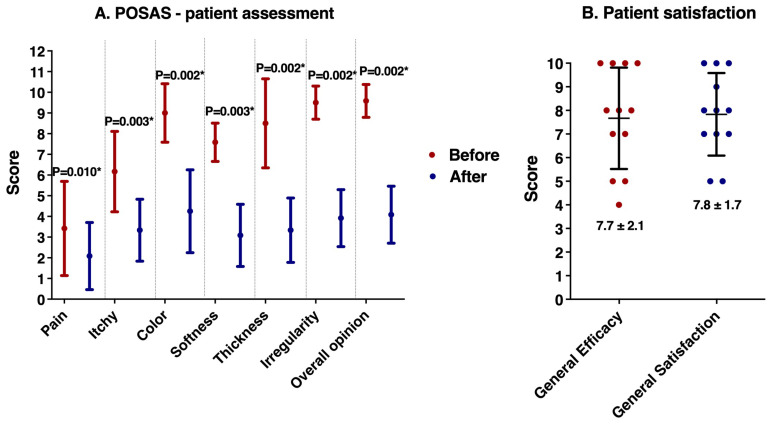
(**A**) The Patient and Observer Scar Assessment Scale (POSAS) measurement based on patient assessments of overall scar conditions before and one year after initial treatment. This measurement is based on a 0–10 scale, with 0 representing no change from normal skin and 10 indicating a large departure. Lower ratings indicated better results. (**B**) Patient-reported satisfaction, which included general comments on the treatment’s efficacy and satisfaction. These measurements were assessed on a scale of −10 to 10, with 10 indicating high satisfaction. * *p* < 0.05 indicates significant difference.

**Figure 3:**
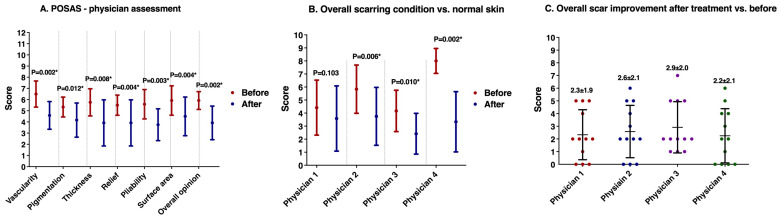
The outcome of the entire treatment. (**A**) The Patient and Observer Scar Assessment Scale (POSAS) measurement based on physician assessments of overall scar conditions before to and one year after initial treatment. This measurement is based on a 0–10 scale, with 0 representing no change from normal skin and 10 indicating a large departure. Lower ratings indicated better results. (**B**) Physician-reported overall scarring condition compared to surrounding normal skin on a 0–10 scale, with 0 representing no difference from normal skin and 10 indicating a severe divergence. Lower scores indicated a better scar condition. (**C**) Physician-reported overall scar improvement six months after initial treatment vs. pre-treatment condition, with scores ranging from 0 to 10 and 10 indicating the most improvement. The dots with different color represented given scores by different physician. Higher scores are preferable. * *p* < 0.05 indicates significant difference.

The POSAS measurement reflects significant improvements in scar appearance following the treatment after one year, as evidenced by the substantial differences in pre- and post-treatment patients’ (Figure 2A) and physicians’ (Figure 3A) assessments. The scoring scale ranges from 0, indicating no difference from normal skin, to 10, which signifies a very large difference from normal skin. Lower scores represent better outcomes, showing minimal deviation from normal skin appearance. It showed a significant improvement in a scar’s pain, itching, color, softness, thickness, irregularity, and distorted appearance after treatment assessed both by the patients and the physicians. Notable enhancements were observed in all aspects of the scar, including vascularity, pigmentation, thickness, relief, pliability, and surface area. 

As shown in Figure 2B, patients rated the treatment’s efficacy highly, with an average score of 7.7 out of 10, signifying marked improvements in scar appearance. Similarly, overall patient satisfaction scored an average of 7.8. These scores indicate that the treatment was not only effective in reducing scar visibility but also met the expectations of patients, resulting in high satisfaction levels.

### 3.3. The Outcome of the Entire Treatment

Figure 3B reveals a consensus among physicians on the overall scarring condition after treatment in comparison with the surrounding normal skin. The results showed significant improvement after treatment was found in these physicians, except for the first. Figure 3C displays the physician’s assessment of scar improvement at six months after the initial treatment vs. the time before the treatment. The measurement was based on a scale of 0 to 10, with 0 denoting no change and 10 denoting a significant improvement. The average scores, which vary from 2.2 to 2.9, point to a generally modest improvement. Interestingly, compared to other physicians, the first physician’s lower average score is consistent with his earlier finding in Figure 3B, suggesting a more conservative assessment of the improvement. Long error bars highlight the variability among individual assessments, reflecting differences in subjective judgment or the unique characteristics of each scar.

### 3.4. Physician Preference for Steroid Cream or Solution after Six Months of Treatment 

**Figure 4:**
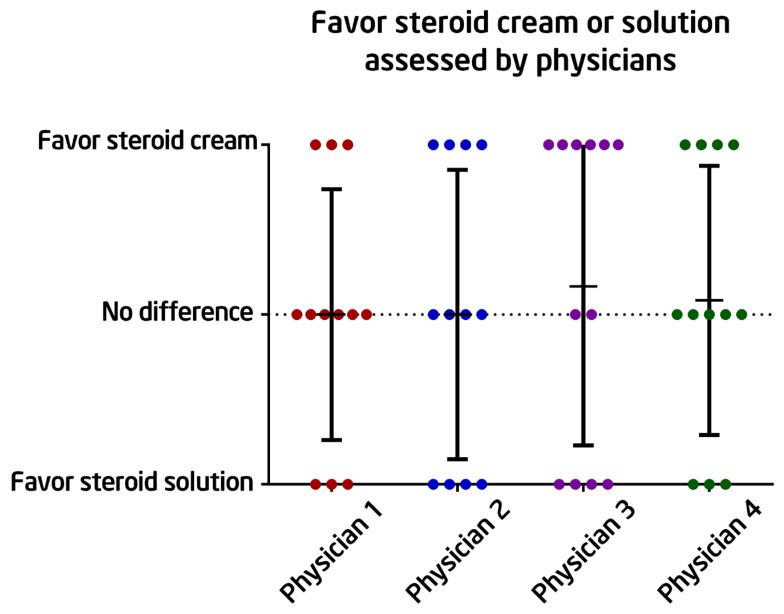
The outcomes were judged using photographs taken after 6 months of initiating treatment and compared to those taken before treatment to determine which side was better than the other. These physicians were blinded to the treatment regimens applied to both sides of the post-thyroidectomy scar. The dots with different color represented given scores by different physician.

Figure 4 depicts the physicians’ preferences for steroid cream and steroid solution. The results were determined by comparing photographs taken after 6 months of treatment to those taken before treatment to determine which side performed better. These physicians were blinded to the treatment regimens used on both sides of the post-thyroidectomy scar. It displays a more diverse collection of results, with some assessments indicating a preference for the cream and others expressing no preference. Overall, there does not appear to be a uniform preference among the physicians, implying that neither the cream nor the solution forms of the steroid showed a significant benefit over the other.

### 3.5. Summary of Results

This study found that fractional CO_2_ laser treatment, combined with either steroid cream or solution, significantly improved scar appearance. However, there is no notable difference in efficacy between the two steroid forms application. Patient satisfaction post-treatment was high, reflecting positively on the treatment’s outcomes. While most physicians agreed on the significant improvement in scar appearance when compared to normal skin and to the scar condition before the treatment, the improvement was only mild.

## 4. Discussion

This study revealed a positive effect of combining fractional CO_2_ laser treatment with topical steroid delivery in enhancing the appearance of post-thyroidectomy scars, showing significant but modest improvements in scar characteristics, high patient satisfaction, and favorable physician evaluations. The fractional CO_2_ laser’s precise and controlled skin resurfacing, which minimizes damage to surrounding tissues, aligns with previous research confirming its effectiveness in scar management and skin rejuvenation [9]. Laser irradiation has also demonstrated significant potential in promoting the regeneration of neuronal tissues through various mechanisms. Studies have shown that pulsed laser microbeam irradiation can create precise zones of neuronal injury, facilitating detailed observations of axonal degeneration and regrowth, and thereby promoting neural regeneration [25]. Low-level laser irradiation has been found to modulate brain-derived neurotrophic factor mRNA transcription via the calcium-dependent activation of the ERK/CREB pathway, enhancing neuronal differentiation and survival [26]. Additionally, the integration of graphene with laser irradiation has demonstrated remarkable potential in enhancing the differentiation of human neural stem cells into neurons, a crucial step for effective neural tissue regeneration. Studies have shown that graphene films, when subjected to pulsed laser stimulation, not only accelerate the differentiation process but also facilitate the formation of organized neuronal networks due to induced thermal gradients [27]. Furthermore, the use of reduced graphene oxide nanomeshes under near-infrared laser stimulation has been found to selectively enhance neuronal differentiation over glial cells, attributed to the beneficial effects of low-energy photoexcited electrons [28]. These findings collectively highlight the promising role of graphene and laser irradiation in advancing neural tissue engineering and the therapeutic promise of laser irradiation in enhancing tissue regeneration and recovery. 

Furthermore, the CO_2_ laser can create microscopic channels that facilitate transdermal drug delivery, while steroids exert anti-inflammatory effects that modulate healing processes. This synergy between thermal tissue injury and pharmaceutical intervention presents a promising scar management strategy. Results indicating notable improvements in color, pliability, and height of scars further corroborate the laser’s utility as a robust treatment option for post-thyroidectomy scar amelioration, consistent with the existing literature on its benefits for scar treatment [29,30,31]. Previously, we had investigated using LADD of topical steroids for treating post-thyroidectomy hypertrophic scars. The research involved ten female patients treated with fractional ablative CO_2_ lasers combined with 0.05% clobetasol propionate, applied monthly, showing significant improvements in scar characteristics which aligned with this study. It highlights LADD’s potential in enhancing topical steroid delivery, effectively reducing scar severity without side effects typically associated with intralesional steroid injections [20]. Similarly, Lin et al. [32] also utilized POSAS to assess the effectiveness of the fractional ablative CO₂ laser combined with topical steroid treatment for post-thyroidectomy hypertrophic scars. This study indicated significant improvements in itchiness, color, softness, thickness, irregularity, and distorted appearance of the scars, although no significant pain relief was reported. Additionally, significant improvements were noted in vascularity, pigmentation, thickness, relief, pliability, surface area, and overall opinion as assessed by treating doctors and based on photographic evaluations by three different doctors. In addition, our findings are consistent with those of Majid et al. [33], who exhibited considerable scar improvement utilizing fractional CO_2_ laser resurfacing paired with strong topical corticosteroids in treating pediatric burn scars. An average 4.2-point decrease in Vancouver Scar Scale scores and excellent physician assessments further validate this approach’s efficacy across different scar types [33], highlighting the potential benefits of laser intervention plus topical steroid for optimizing scar outcomes.

In this study, no significant difference emerged between using steroid cream or solution formulations with laser treatment. Creams are emulsions that absorb into the skin without leaving residue, making them suitable for dry or inflammatory conditions due to their moisturizing effects. Solutions, on the other hand, are alcohol based, less greasy, and ideal for hairy areas as they spread easily and dry quickly [19]. In treating the neck area, cream formulations may be more suitable than solutions as creams offer superior moisturizing properties essential for healing and minimizing scarring, which tends to be less irritating for sensitive neck skin. Additionally, creams are easier to apply with controlled dosing compared to solutions. However, notably, a randomized trial conducted by Glees et al. [34] tested the efficacy of 1% hydrocortisone cream versus 0.05% clobetasone butyrate cream in treating radiation dermatitis. Despite receiving identical radiation doses, patients using clobetasone butyrate cream had more severe radiation reactions than those using hydrocortisone cream. These findings highlight the crucial role of steroid formulation and concentration in determining the efficacy and tolerability of topical steroid treatments. The choice between these formulations should consider factors such as the nature of the skin condition, the treatment area’s characteristics, and patient preferences to ensure optimal efficacy and comfort. For post-thyroidectomy scars, cream formulations may be more suitable as they offer superior moisturizing properties essential for healing and minimizing scar appearance, and tend to cause less irritation for sensitive neck skin.

A study performed by Lin et al. [32] revealed that applying 0.05% clobetasol propionate ointment following fractional CO_2_ laser treatment did not effectively prevent the formation of a hypertrophic scar immediately after thyroidectomy. The authors suggest that the timing of steroid application may not have aligned with the optimal window for modifying the scar formation process. During the initial stages of scar formation, there is significant macrophage infiltration and cytokine secretion, which may not be effectively inhibited by the low-dose glucocorticoids used. Glucocorticoids may be more effective at reducing fibroblast proliferation and inducing apoptosis during the latter, static stage of scar formation [32]. The lack of efficacy could stem from a mismatch between the biological activity at the treatment time and the pharmacodynamics of the steroids. Other studies indicate that the skin barrier function largely recovers within 12–16 h post-fractional CO_2_ laser treatment, influencing the effectiveness of transdermal drug delivery, such as topical steroids [35,36]. The quick repair of the skin barrier most likely explains why this study found no significant difference in the efficacy of steroid creams versus solutions. For this study, both formulations had a similarly brief window for optimal absorption due to the quick healing post-laser treatment. Exploring methods to prolong skin permeability, such as using penetration enhancers or occlusive dressings, may potentially enhance the effectiveness of different topical steroid formulations with LADD.

Importantly, potential side effects of the combined fractional CO₂ laser and topical steroid treatment for post-thyroidectomy scars include temporary redness, swelling, and discomfort at the treatment site, which typically resolve within a few days. There is a risk of infection if proper post-treatment care is not followed, although this is rare with appropriate hygiene practices. Some patients may experience changes in skin pigmentation, such as hyperpigmentation or hypopigmentation, particularly those with darker skin tones. While topical corticosteroids have seemed to be effective in scar treatment, their overuse is a substantial clinical problem. Prolonged use of topical steroids can lead to skin thinning, atrophy, or telangiectasia, although these effects are minimized by the limited and localized application in this treatment protocol. Rarely, patients might develop an allergic reaction to the topical steroids or other components used during the procedure. Rathi et al. [37] advise against widespread usage, which can result in major adverse effects including atrophy, rosacea, and tachyphylaxis. Meena et al. [38] observed that of 85,280 patients examined in one year, 370 (0.43%) had adverse effects due to topical corticosteroid misuse. This study found that tinea incognito was the most prevalent side effect, affecting around 50% of patients, followed by acne in around 30% of patients. These side effects were mostly caused by the excessive and improper overuse of potent topical corticosteroids, specifically clobetasol propionate, as reported by 44.33% of patients experiencing unfavorable symptoms. The LADD of the steroid into the scar within a limited time of treatment may help reduce the possible complications associated with repeat topical steroid use.

Post-thyroidectomy scar recurrence can be influenced by individual healing characteristics, genetic predisposition, and environmental factors. Studies suggest that the effects of laser treatments may diminish over time, leading to partial recurrence. Early postoperative treatment with fractional carbon dioxide lasers or 1550 nm fractional erbium-glass lasers has shown efficacy in reducing scar visibility, with better outcomes when initiated soon after surgery [29,39]. The addition of topical steroids may prolong treatment effects by modulating the inflammatory response and collagen production, significantly improving scar outcomes and reducing recurrence [20,40]. These combined approaches highlight the importance of early and integrative treatments in managing post-thyroidectomy scars for better long-term cosmetic outcomes.

While this study was performed based on the prospective, randomized, and split-body study and revealed a positive effect of combining fractional CO_2_ laser treatment with topical steroid delivery in enhancing the appearance of post-thyroidectomy scars, this study has several limitations that should be considered when interpreting the results. First, the sample size was small, with only 12 patients included, which may limit the generalizability of the findings. Second, this study was conducted at a single center, potentially introducing site-specific biases and limiting the applicability of the results to broader populations. Third, the lack of a control group without laser and/or steroid treatment means that we cannot fully ascertain the independent effects of the treatments. Additionally, the follow-up period was relatively short, preventing us from assessing long-term outcomes and the possibility of scar recurrence. Finally, lifestyle factors such as diet, smoking, and sun exposure, which could influence treatment effectiveness, were not controlled for or systematically recorded, potentially affecting the variability in treatment responses. Larger multi-center studies with more diverse populations and extended follow-up periods are necessary to fully understand the efficacy, safety, and patient satisfaction associated with the combined use of fractional CO_2_ laser and topical steroids in scar management.

## 5. Conclusions

This prospective, randomized, split-body study demonstrated that combining fractional CO_2_ laser treatment with topical steroid delivery, either cream or solution form, significantly improved the appearance of post-thyroidectomy scars. Both approaches achieved enhancements in scar characteristics like vascularity, pigmentation, and thickness compared to pre-treatment, with high patient satisfaction. Importantly, no significant difference emerged between utilizing steroid cream versus solution formulations when coupled with laser therapy. Overall, the findings indicate fractional CO_2_ laser plus topical steroids represents a promising non-invasive strategy for post-surgical scar management, though further large-scale, long-term studies evaluating ideal formulations, dosing, and safety are warranted.

## Data Availability

The original contributions presented in this study are included in the article, further inquiries can be directed to the corresponding author.
